# Using Rasch Analysis to Assess the Psychometric Properties of a Five-Item Version of the General Self-Efficacy Scale in Adolescents

**DOI:** 10.3390/ijerph19053082

**Published:** 2022-03-06

**Authors:** Anne Mari Steigen, Hanne Søberg Finbråten, Annette Løvheim Kleppang

**Affiliations:** 1 Department of Health and Nursing Sciences, Faculty of Social and Health Sciences, Inland Norway University of Applied Sciences, P.O. Box 400, 2418 Elverum, Norway; hanne.finbraten@inn.no; 2 Department of Public Health and Sport Sciences, Faculty of Social and Health Sciences, Inland Norway University of Applied Sciences, P.O. Box 400, 2418 Elverum, Norway; annette.kleppang@inn.no

**Keywords:** adolescents, general self-efficacy scale, psychometrics, Rasch analysis, self-efficacy, validation

## Abstract

The aim of the present study was to use Rasch analysis to assess the psychometric properties of the five-item version of the General Self-Efficacy Scale (GSES) amongst adolescents aged 13 to 19. In this cross-sectional study, 6265 adolescents responded to a web-based questionnaire. Data collected from lower and upper secondary schools in Norway, during 2018, were analysed using the partial credit parameterisation of the unidimensional Rasch model. The five-item version of the GSES was found to be unidimensional and to have acceptable reliability. The targeting of the scale could have been better. All items had ordered thresholds, indicating that the response categories worked quite well. The five-item version of the GSES has potential for measuring self-efficacy in a general population of adolescents. In surveys concerning adolescents’ mental health, it is important to include aspects of positive mental health and health-promoting factors, such as self-efficacy.

## 1. Introduction

The developmental stage, which adolescence represents, can be a challenging period for many adolescents undergoing major psychological, physical, and social changes. This period can be especially challenging for adolescents experiencing psychosocial risk factors [1]. At the same time, this transactional period provides the opportunity to foster adolescents’ strengths and thereby enhance positive outcomes [1,2]. Hence, there is a need for more knowledge not only about risk factors and challenges in adolescents but also regarding factors that can potentially be a strength and buffer against stress and other risk factors. This is in accordance with the salutogenic model, which views health as a movement on a continuum between ease and dis-ease and focusses on the factors that promote movement towards the healthy end of the continuum [3].

Self-efficacy refers to a person’s belief in their ability to master challenges and everyday life in general. The concept was originally developed by Bandura [4] as part of his social-cognitive theory. Developing and strengthening self-efficacy in adolescents is emphasised as important and may influence which course the adolescents’ life paths take [5]. Hence, self-efficacy is seen as a positive psychosocial factor, in contrast to negative psychosocial factors, such as stress and loneliness, and might have a buffer role for negative psychosocial factors [6]. 

Moreover, self-efficacy is considered an important general health promotion factor for both physical and mental diseases, and it is essential when working with health promotion in school settings [7]. Self-efficacy may also positively influence adolescents’ physical activity [8], risk-taking behaviour, and health decisions [9]. Another study has found that self-efficacy could be crucial for adolescents’ perception of life satisfaction and their capacity to cope with normative stressors [10]. Studies measuring self-efficacy have shown that girls score significantly worse on self-efficacy than boys [6,11]. However, Jerusalem and Hessling [7] have concluded that self-efficacy can be systematically strengthened in school through interventions designed to promote it. 

Bandura (4) originally described self-efficacy as related to specific competences and skills and not to coping in general, meaning that self-efficacy is situation-specific. Thus, it is possible to have high self-efficacy in one area and low in another. This has resulted in several self-efficacy scales related to specific areas, such as breastfeeding [12], exercise, pain, and so forth [13]. However, other researchers [14,15] have argued for a more generalised concept of self-efficacy, wherein the most important contributing factor is experiences of mastering [15]. Experiencing failure or success in various situations could result in generalised self-efficacy. Therefore, different generalised self-efficacy scales have been developed, one of which is Schwarzer and Jerusalem’s [15] General Self-Efficacy Scale (GSES), which is widely used and has been translated into numerous languages [16,17]. 

The original GSESconsists of 10 items. In population-based studies, psychometric instruments, in their original form, are often too lengthy to be suitable. Therefore, short versions are constructed [17]. A five-item short version of the GSES is used in the Norwegian Municipal Youth Surveys, a national general population study amongst adolescents in Norway. This five-item version of the GSES has also been employed in the Norwegian mother and child study (MoBa study) [17]. The five-item version was developed using stepwise multiple linear regression analysis on population-based data among 18-year-olds [17]. A sum score of the five items in the short version correlated 0.96 with the full version, and Cronbach’s alpha for the five-item version was 0.78. In MoBa data, the Cronbach alpha was 0.84 [17]. 

Previous psychometric analyses of the GSES have mainly utilised classical test theory [18], although a few studies have used Rasch analysis when the instrument was applied to various populations [19,20]. To our knowledge, only one study has tested the original 10-item GSES applied to adolescents using Rasch analysis [20]; it was based on data from 2009 and 2010 amongst adolescents aged 13 to 15. 

Except for the description by Tambs and Røysamb [17] about the development of the five-item version of the GSES, there are, to our knowledge, no studies assessing the psychometric properties of this shortened version. Furthermore, there is a lack of studies exploring the psychometric properties of this version when applied to adolescents. 

It is crucial that policy and practise are based on data from healthcare research using reliable and valid scales. The purpose of this study is, therefore, to assess the psychometric properties of the five-item version of the GSES, used in the Municipal Youth Surveys in Norway, by means of Rasch analysis. 

## 2. Materials and Methods

This study is based on data from the Municipal Youth Surveys (Ungdata Survey) [21], conducted by the Norwegian Social Research (NOVA) at Oslo Metropolitan University, in collaboration with all Regional Drug and Alcohol Competence Centres (KoRus). The Municipal Youth Surveys represent an ongoing, representative cross-sectional study of adolescents, from grades 8 to 13, across almost all municipalities in Norway. The Municipal Youth Surveys are financed, partially, by the Norwegian Directorate of Health and cover different aspects of adolescents’ lives (e.g., health issues, local environment, school issues, lifestyle behaviours, relationships with friends and parents, symptoms of depression, and self-efficacy). 

In this study, data from the survey conducted in 2018 were used. Parents and adolescents were informed by email in advance; parents of adolescents aged 13 to 17 were assured that they could withdraw their children from participation at any time, and adolescents were informed that participation was voluntary. The study was conducted as a web-based questionnaire administered at school during school hours with a teacher or an administrator present to help the participants if they had any questions. The surveys consist of a mandatory part, which is similar in all participating municipalities, in addition to a set of predefined optional questions from which municipalities could choose, based on interest and need. The GSES is from the optional part. 

The adolescents used approximately 30 to 45 min to complete the questionnaire. Altogether, 6265 adolescents, aged 13 to 19, who answered all the self-efficacy questions were included in our analyses. The analysis was, therefore, based on complete data. Independent researchers, who did not participate in data collection, managed and analysed an anonymous data file. The study was conducted in line with the Declaration of Helsinki, and the Norwegian Centre for Research Data (NSD) approved all privacy aspects of the study. 

Self-efficacy was measured using five items derived from the 10-item version of the GSES [15,17]. The adolescents were asked how true the following statements are according to their experience: ‘I always manage to solve difficult problems if I try hard enough’ (item 1); ‘If someone opposes me, I can find the means and ways to get what I want’ (item 2); ‘I am confident that I could deal efficiently with unexpected events’ (item 3); ‘I can remain calm when facing difficulties because I can rely on my coping abilities’ (item 4); ‘If I am in trouble, I can usually think of a solution’ (item 5). The five-item version of the GSES has four response categories: not at all true (1), hardly true (2), moderately true (3), and exactly true (4).

The partial-credit parameterization (PCM) [22] of the unidimensional Rasch model [23] was applied due to a significant Likelihood ratio test (Chi Square was 204.04, df 7, with a probability <0.001). This model was used to examine whether the five-item version of the GSES meets the requirements of measurement such as local independence, invariance, and proper response categorisation. This shortened version of the GSES was examined at both a general level and finer level of analysis. The person separation index (PSI), which could be considered analogous to Cronbach’s alpha [24], was used as an indicator of reliability. Local independence implies that there should not be any further relationship amongst the items than the latent trait [25] (here, self-efficacy), which could be violated by multidimensionality or response dependence. 

To explore dimensionality of the five-item GSES, the procedure of combined principal component analysis (PCA) of residuals and paired *t*-tests was used. Based on the PCA, two subsets of the scale were made, and the person estimates for the two subsets were compared using paired *t*-tests. Multidimensionality could be considered evident if the proportion of individuals with significantly different person-location estimates on the pair of compared subscales exceeds 5% [26,27]. A residual correlation of <0.3 was applied as an indicator of response dependency. Targeting was examined by comparing the items and person locations, and a scale is considered well targeted if the mean person location values are around 0 [28]. Targeting was also explored graphically. 

To analyse item fit, chi-square statistics and standardised residuals, based on comparisons between observed and expected values, were used. Fit residuals in the range ±2.5 indicate adequate item fit, and chi-square probability values above Bonferroni’s adjusted 5% suggest adequate item fit [28]. Item characteristic curves (ICCs) were inspected to assess item fit graphically. To examine whether response categories were working as intended, the threshold ordering was inspected both statistically and graphically. 

For comparisons of scores across groups to be valid, the measurement scale must be invariant. Items displaying differential item functioning (DIF) across sample groups indicates lack of invariance. The items were analysed, with respect to DIF, for the person factors gender, school level, and grade. In addition to inspecting graphical displays, two-way analysis of variance (ANOVA) of standardised residuals was employed to detect the DIF [29]. Statistical significance was assumed at a Bonferroni-adjusted 5%. 

Significance tests are sensitive to sample size. As this study included a rather large sample size, there was a risk of drawing false conclusions [30]. In such situations, Bergh [31] recommends performing analyses in random subsamples to avoid potential bias. Sample size was calculated by multiplying the number of items (5) by the number of thresholds (3), with 30 individuals per threshold [32], yielding a sample size of 450 (5 × 3 × 30), which can be deemed as adequate in these analyses. Hence, 10 randomly selected subsamples of 450 were drawn for further analyses concerning item fit and DIF. All analyses were performed using the software RUMM2030Plus (RUMM Laboratory Pty Ltd., Perth, Australia) [33].

## 3. Result

About two-thirds of the respondents were recruited from upper secondary schools (Table 1). The sample comprised an approximately equal proportion of males and females.

### Rasch Analysis

**Figure 1:**
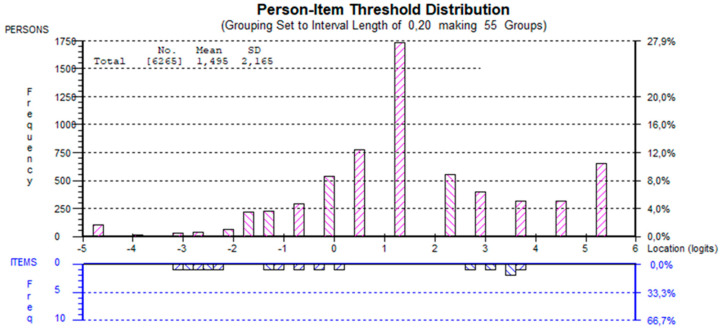
Distribution of person estimates (above the *x*-axis) and item threshold estimates (below the *x*-axis). Results are based on complete data (n = 6265).

The five-item version of the GSES formed a unidimensional scale (significant *t*-tests 4.90% [total sample], 4.82% [lower secondary school], 4.51% [upper secondary school]) with sufficiently high reliability (PSI: 0.79 and Cronbach’s alpha: 0.88 [total sample]; PSI and Cronbach’s alpha for lower secondary schools were 0.80 and 0.88, respectively, and for upper secondary schools, 0.78 and 0.88, respectively]). The targeting of the scale could have been better (mean person location estimate: 1.49 logits [total sample], 1.45 logits [lower secondary school], and 1.52 logits [upper secondary school]). There were no items covering the location between 0.5 and 2.5 logits, where most respondents were located (Figure 1). Moreover, the person-item distribution indicates extreme values at the upper end. The targeting was better for females (mean person location estimate: 1.11 logits [total sample]) compared to males (mean person location estimate: 1.91 logits [total sample]). There were no signs of response dependency.

**Figure 2:**
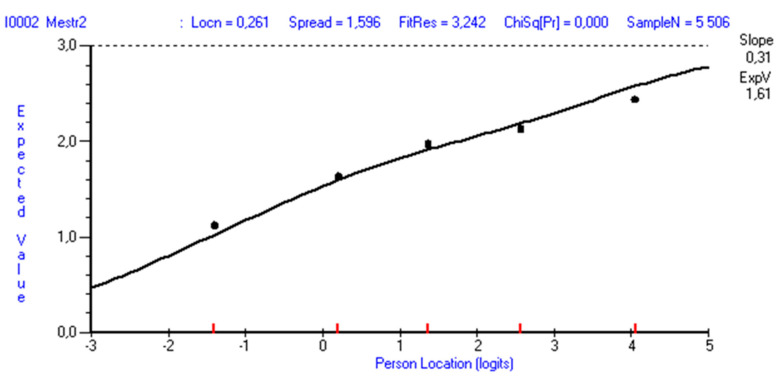
Item characteristic curve for item 2 (If someone opposes me, I can find the means and ways to get what I want). Results are based on complete data (n = 6265).

In the full sample, all items displayed significant misfit (Table 2), but this was not evident when sample size was reduced to 450. Item 2 tends to under-discriminate in the total sample (fit residual 3.242), whereas items 3, 4, and 5 over-discriminate (fit residuals: −14.648, −7.792, and −10.918, respectively). Drawing 10 random samples of n = 450, item 2 displayed significant misfit in four of them, but the fit residuals were within the recommended range of ±2.5 (varying between 1.182 and 1.900). Inspecting the ICCs, the item seems to work quite well (Figure 2). The response categories also work well for all items. Items 1 and 5 were the easiest to endorse, whereas item 4 was the most difficult.

**Figure 3:**
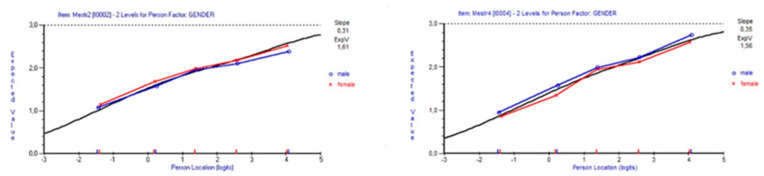
Graphical comparison between means of males and females, in five class intervals, for items 2 (If someone opposes me, I can find the means and ways to get what I want) and 4 (I can remain calm when facing difficulties because I can rely on my coping abilities). Results are based on complete data (n = 6265).

Considering the whole sample, items 1, 2, and 4 displayed statistically significant DIF regarding gender, with items 1 and 2 favouring females and item 4 favouring males. Examining the ICCs, the DIFs seem marginal. Drawing 10 random samples of n = 450, DIF for gender was evident for item 2 in two random samples, whereas gender DIF was evident for item 4 in three random samples (Figure 3).

The same was evident when analysing lower and upper secondary schools separately. None of the items displayed DIF for school level or grade (when analysing upper and lower school separately) in any of the sample sizes.

## 4. Discussion

This study was the first to use Rasch analysis to assess the psychometric properties of the five-item version of the GSES amongst adolescents. The scale displays satisfactory psychometric properties at an overall level. It was found to be unidimensional and have acceptable reliability. However, the targeting could have been better. At first, the items could seem to have a relatively poor fit to the Rasch model. However, when adjusting for sample size and inspecting the graphical displays, the items seem to work quite well.

Inspecting the item location, relative to the respondents’ locations, the five-item version of the GSES was somewhat out of target, meaning that the adolescents have higher self-efficacy than the scale can measure. We have not seen other studies examining the psychometric properties of this five-item version. However, the targeting of this version is in line with that of a 10-item version of the GSES applied amongst Swedish adolescents [20]. Bad targeting implies that the precision of the scale becomes poorer because it lacks items for certain degrees of difficulty, which could be compared to measuring parts of something by centimetres and other parts in inches. 

The item locations could be considered quite close. Hence, some of the items should be reworded to increase their difficulty, as well as the scale’s ability, to differentiate between people with lower and higher proficiency. When item thresholds are more widespread, there is a better ability to separate people along the latent trait, which might also increase the reliability of the scale. The scale was better targeted to females than males. This is in line with previous studies of Norwegian adolescents, which reported lower self-efficacy amongst females compared to males [6,11].

Regarding the chi-square statistics for the five-item version of GSES, all items displayed significant misfit. One item (item 2, If someone is opposing me, I can find methods and ways of getting what I want) tended to under-discriminate, and three items over-discriminated. Under-discriminating items tend to overlap with other constructs that are not correlated with the latent trait [34]. Given the large sample size, inspecting the graphical display of the ICC is also important to judge the magnitude of the misfit. When items show misfit in the formal test statistics, but the graphical display shows that the observations are located close to the expected values, the misfit could be considered as only minor [24]. This is confirmed when performing repeated analyses for 10 random samples of n = 450. However, the reason for this item tending to under-discriminate could be due to a translation error. Item 2 has also been discussed as problematic in other studies [19,20], being the only one to include an interpersonal aspect. According to Bandura [4], this item might reflect another source of information, which impacts a person’s ability: verbal persuasion. If someone opposes a person, this could involve discouraging words or hostile actions.

The response categories worked quite well. However, the third response category (moderately true) was most often chosen by the respondents, which implies that this category was relatively wide. Hence, the scale could benefit from offering additional response categories, which might increase the reliability index as more measuring points are added [35]. 

In addition, the Norwegian translation could be considered as deviating from the original wording, as the response categories are more polarised in Norwegian (from very wrong to very true), whereas the original wording reflects levels of truth. Categories reflecting levels of truth also seem to give higher variance than the Norwegian wording of the response categories used in the present study (Røysamb 2021, personal communication). Hence, comparisons of scores across studies using different response categories are not recommended, as this might result in differences in average scores (Røysamb 2021, personal communication). 

Drawing 10 random samples of n = 450, DIF was only present in 2 of 10 subsamples for item 2 and 3 of 10 subsamples for item 4. Hence, DIF could be considered as not being a threat for comparison of self-efficacy scores across gender or school level based on this five-item version of the GSES. This is in contrast to the study by Lönnfjord and Hagquist [20], who found statistically significant DIF for item 4 (item 6 in the 10-item version), while also considering a reduced sample size. 

The main strength of this study is the use of a modern test theory approach, Rasch analysis, to assess the psychometric properties of the five-item version of the GSES. Short versions with strong psychometric properties are favourable for use in large population-based studies. 

This study includes a large sample size, covers data from the whole country, and provides an updated description of adolescents’ responses to a five-item version of the GSES. However, the analyses were based on self-reported data; hence, there might be a risk for response bias (e.g., social desirability). Nevertheless, considering the large sample size of this study and the fact that the questionnaire was completed anonymously, the potential random errors might be minimised [36]. 

## 5. Conclusions

Overall, the five-item version of the GSES works well when applied to Norwegian adolescents. Requirements for measurement, such as local independence and invariance, were met, and the scale has acceptable reliability (PSI). The targeting of the scale could probably be strengthened by rewording the Norwegian labelling of response categories. Nonetheless, all five items in the present version of the GSES displayed an acceptable fit to the Rasch model. Hence, this scale is suitable for measuring self-efficacy in a general population of adolescents. 

More knowledge about health promoting factors, such as self-efficacy, is important to strengthen mental health and prevent mental health problems in adolescents. Further, strengthening mental health in adolescence might help with building robustness in adulthood. In surveys concerning adolescents’ mental health, it is, therefore, important to include aspects of positive mental health and health-promoting factors, such as self-efficacy. The five-item version of GSES is suitable for this purpose.

Future studies should focus on factors potentially strengthening or influencing self-efficacy in adolescents. This knowledge is important for both national and local public health workers and can guide the implementation of health-promoting strategies and interventions for adolescents. 

## Figures and Tables

**Table 1:** Sample characteristics (n = 6265) of the participants in the Municipal Youth Survey from 2018.

Characteristic	n (%)
Gender	
Male	3017 (48)
Female	3201 (51)
Missing	47 (1)
Education	
Lower secondary school	2159 (35)
Grade 8	626 (29)
Grade 9	746 (35)
Grade 10	654 (30)
Missing	133 (6)
Upper secondary school	4106 (66)
Year 1	1649 (40)
Year 2	1404 (34)
Year 3	1047 (26)
Missing	6 (0)

**Table 2:** Item fit statistics for the five-item version of General Self-Efficacy Scale based on the Municipal Youth Surveys from 2018 (n = 6265).

		Total Sample				Lower Secondary School				Upper Secondary School			
Item	Label	Loc.	Fit Resid.	χ^2^	Prob. *	Loc.	Fit Resid.	χ^2^	Prob. *	Loc.	Fit Resid.	χ^2^	Prob. *
1	I always manage to solve difficult problems if I try hard enough.	−0.340	0.359	147.047	<0.001	−0.349	−0.919	57.863	<0.001	−0.336	1.105	89.723	<0.001
2	If someone opposes me, I can find the means and ways to get what I want.	0.261	3.242	115.717	<0.001	0.291	3.594	46.927	<0.001	0.240	1.230	92.762	<0.001
3	I am confident that I could deal efficiently with unexpected events.	−0.039	−14.648	141.073	<0.001	−0.010	−6.320	42.311	<0.001	−0.058	−13.701	96.426	<0.001
4	I can remain calm when facing difficulties because I can rely on my coping abilities.	0.464	−7.792	85.278	<0.001	0.398	−2.930	20.711	0.001	0.506	−7.568	67.049	<0.001
5	If I am in trouble, I can usually think of a solution.	−0.347	−10.918	104.567	<0.001	−0.331	−4.889	25.708	<0.001	−0.352	−10.037	67.694	<0.001

* Bonferroni-adjusted 5%. df for items = 5, Fit resid. = Fit residual; Loc = Location; Prob = Chi square probability.

## Data Availability

Data and materials in the Ungdata surveys are included in a national database administered by Norwegian Social Research (NOVA). Data are available for research purposes upon application. Information on the questionnaires can also be found on the webpage (in Norwegian) (http://ungdata.no/) Accessed on 15 November 2021.
